# Requirements and Value Elicitation for a High-Fidelity Pelvic Floor Simulator for Physiotherapists: Mixed Methods Study

**DOI:** 10.2196/72119

**Published:** 2025-05-15

**Authors:** Yael Zekaria, Antonia Tzemanaki, Jonathan Rossiter

**Affiliations:** 1 School of Engineering Mathematics and Technology, University of Bristol, Ada Lovelace Building, Bristol, BS8 1TW, United Kingdom, 44 117 928 9000; 2 School of Engineering, University of the West of England, Bristol, United Kingdom; 3 Bristol Robotics Laboratory, Bristol, United Kingdom

**Keywords:** pelvic examination, high-fidelity simulation, simulation-based learning, medical education, medical devices, physiotherapy

## Abstract

**Background:**

Physiotherapists lack training opportunities for repeated practice of pelvic examinations for the identification of pelvic floor disorders (PFDs), leading to low confidence in the clinical setting. Pelvic simulators exist and are a valuable supplement to the medical curriculum, yet none demonstrate pelvic floor muscle (PFM) function or dysfunction. To design effective simulators, an assessment of end-user requirements is essential.

**Objective:**

This study aimed to elicit physiotherapists’ needs and requirements for a high-fidelity PFM simulator and the associated use cases.

**Methods:**

This study followed a mixed methods design by collecting qualitative and quantitative data from a web-based survey. Quantitative data were analyzed using descriptive statistics and differences between demographic groups were calculated using 2-sample Kolmogorov-Smirnov 2-sided tests. Qualitative data were analyzed using thematic analysis.

**Results:**

In total, 66 physiotherapists completed the survey. The most common suggested use cases of the simulator were for training and professional development (56/66, 84.9%), and patient education (48/66, 72.7%). Pelvic organ prolapse and muscle tone function and dysfunction were identified as the most useful PFDs for the simulator to demonstrate. Positional tracking and force sensing were considered important features and there was a preference for a generic over a pathology-specific or patient-specific simulator. A total of 3 themes emerged through the qualitative analysis: prioritizing patient care; representing the variability in anatomy and PFDs for simulator realism; and consideration of the implementation, cost, and accessibility of simulators.

**Conclusions:**

There is value in PFM simulators for physiotherapists for multiple use cases. Design recommendations include using realistic materials, demonstrating PFM dynamics, modularity to vary the complexity for different end-users, offering a range of feedback modalities for position and pressure sensing, and ensuring affordability and curriculum integration.

## Introduction

### Background

Over 60% of people with female-at-birth anatomy experience symptoms of pelvic floor disorders (PFDs) [1] such as incontinence, pelvic organ prolapse (POP) and pelvic pain. In the first course of treatment for PFD symptoms, it is recommended to contact a physiotherapist who will perform a pelvic examination to assess the pelvic floor musculature [2]. Most physiotherapists learn examination skills through peer practice which provides a way to teach ethical consent and allows students to practice their skills on each other’s bodies before a patient [3]. However, peers may not present with any PFDs and the idea of being examined by a peer can be uncomfortable for some who if they choose to opt out, miss this opportunity to develop their clinical skills. As a result, it is difficult to gain experience diagnosing and assessing the numerous PFDs.

Physical simulators play a valuable role in supporting obstetrics and gynecology education and training, from offering visualization of anatomy to refining examination skills through repeated practice [4]. While a range of pelvic simulators are on the market, none represent the dynamics of the pelvic floor muscles (PFMs) to the degree of fidelity required to transfer knowledge and skills from the classroom to the clinic.

In a systematic review of simulation training for pelvic examination, simulation-enhanced education was found to improve students’ technical skills, comfort and communication with the patient. However, where offered, the most effective training method is by Gynecological Teaching Associates (GTAs) or standardized patients, volunteers with whom students can practice hands-on examination techniques [5]. As supplements to the medical curriculum, simulation-based teaching has proven effective in improving technical and communication skills for physiotherapists specifically [6]. Simulators can uniquely offer the opportunity for repeated practice and incorporate necessary proprioceptive, visual and tactile feedback to enhance the practitioner’s motor control without risk of harm to patients, GTAs or standardized patients [7].

In simulation, (engineering) fidelity is defined as the extent to which the appearance and behavior of a simulator resembles reality, whereas realism, or psychological fidelity, refers to the authenticity of the scenario in which the simulator is used [7,8]. More recently, fidelity in simulation-based education has been subdivided into the following four elements [8]: (1) environmental element - the training facility and equipment used. (2) Patient element - the virtual reality, full body or part-task trainer used to represent the patient. (3) Semantical element - the scenario in which the simulator is used and the associated intention for the user. And (4) phenomenal element - the engagement of the user through their emotional and cognitive experiences while involved in the simulation activity.

It is commonly agreed that the levels of fidelity and realism of a simulator are dependent on the skills it is being used to teach, and for whom it is intended [4,7-9]. Accordingly, the first stage to developing simulators is identifying the skill or procedure that must be learned or demonstrated [7,9,10]. Engaging with end-users is the only reliable way to ascertain these skills and subsequently, the relevant design requirements needed to teach them [9,11].

Most commercial pelvic simulators focus on childbirth, pelvic examination and surgical skills [12]. To the authors’ knowledge and at the time of writing, no commercial simulator demonstrates any PFD or even the difference between increased, normal and reduced muscle tone. Decreased or reduced tone and incorrect functioning of the PFMs are some of the main causes of PFDs [13].

### Previous Work

Although simulators for certain PFDs have been explored in the research literature, their fidelity and validity vary. In addition, it is rare to describe the design requirements or user needs which would support the future of simulation-based education in pelvic health care. In one of the few simulators designed to mimic the PFM activity for practicing digital examination, Saleme et al [14] modelled the levator ani by 5 springs attached at the relevant points on a resin bony pelvis model. The modified Oxford scale was simulated by varying the spring tension and a physiotherapist identified most differences between the scale points by digitally palpating a specified site between the springs [14]. While this simulator was shown to be a complementary tool to the curriculum, its fidelity was limited by the use of nonrealistic materials. These examples show that both high- and low-fidelity simulation can be effective in medical education, especially for visualizing the 3-dimensionality and dynamics of PFM function and for building confidence before examining patients.

A dynamic and high-fidelity POP simulator was shown to significantly improve the understanding and confidence in performing the pelvic organ prolapse quantification (POP-Q) examination [15]. This simulator was designed such that a user can manually invert a silicone vaginal canal to different extents and angles to simulate anterior, posterior, and apical prolapse. Although medical students and residents at all levels of training were included in this study, responses from all participants’ pre- and post-tests were averaged. In another study it was shown that medical students preferred a low-fidelity sock and tube model in their first and second years of residency compared to third and fourth-year residents who had neutral attitudes toward the utility of the model and preferred conventional learning modalities [16]. In an attempt to make what can often be an awkward part of medical training more lighthearted, a Santa Claus hat was used in another study to visualize and simulate POP-Q but the model itself was not evaluated with any meaningful metrics [17]. Through a robust Delphi study, Meyer et al [18] developed a list of criteria for a 3D pelvic model for anatomy and physiology education. While a range of experts from different disciplines were consulted and the methodology was validated rigorously, no physiotherapists were included in the study, the model in question was computer-based and was neither intended for clinical examination nor to have dynamic features. Physical 3D pelvic models and simulators described in the literature tend to be co-designed by medical professionals working in pelvic health [19-22] which may indicate why no user requirements have been published previously. In the evaluation of the 3D-printed model by Kiesel et al [23], medical students reported that the option to remove parts of the anatomy and parts being printed in different colors was helpful for their understanding of the anatomy. Students also found it useful to be able to angle the model in upright and tilted positions and would approve of models that simulate certain medical conditions and operations.

Although the aforementioned examples show positive efforts toward the use of simulation for teaching pelvic examination skills, they highlight the need for more advanced methods, incorporating higher levels of fidelity for more effective training as seen in numerous soft robotic organ models in the literature [24]. This need is even more pronounced considering that veterinary students practice palpation and even the assessment of pain symptoms in the pelvic region of cows and horses using simulators enhanced with haptic feedback [25,26] whereas pelvic physiotherapy training for humans with female-at-birth anatomy continues to rely on peer examination.

The research in this area shows an appetite for high-fidelity simulation training in the gynecological curriculum with a lack of investigation into the specific needs of physiotherapists who are one of the main treatment providers for people with PFDs.

### Aims of This Study

In this study, we aimed to explore physiotherapists’ perceptions of the value, requirements and potential use cases of a high-fidelity PFM simulator for female-at-birth anatomy. Through a mixed methods survey, we sought to elicit the design recommendations for such a simulator and gain a deeper understanding of the motives behind these preferences.

## Methods

### Survey Development

We took a mixed methods approach by collecting qualitative and quantitative data through an internet-based survey. The quantitative component, which comprised the majority of the survey, informed the preferences of certain features and functionalities of the simulator. The qualitative component was intended to gather a deeper understanding of how a simulator can be integrated into pelvic health education and research, along with the associated challenges.

The survey consisted of 10 closed-ended (quantitative) and 3 open-ended (qualitative) questions. The complete questionnaire is shown in Multimedia Appendix 1. Up to 3 additional open-ended questions were asked to respondents who selected certain choices from three of the closed ended questions to better understand their choices. Respondents had the opportunity to add any further comments at the end of the questionnaire and provide a contact email if they wished to be contacted for an informal follow-up interview. Questions on professional experience and demographics were asked at the beginning and end of the survey, respectively.

Closed-ended questions formed the quantitative component of the survey and concerned the features of the simulator. These questions were formulated after exploring state-of-the-art commercial (Clinical Female Pelvic Trainer; Limbs and Things, Bristol, and United Kingdom) and research-based [15,20,27] simulators. We compiled potential use cases, target anatomy, and any additional features into multichoice, ranking and rating scale questions.

The rating scale questions varied from 3- to 5-point scales to reduce the chance of respondents becoming fatigued which can discourage survey completion and result in satisfyingbehavior [28]. The number of scale points was chosen depending on the most appropriate level of granularity for each item that would provide a meaningful understanding of the respondents’ opinions [28]. Where relevant, closed-ended questions included optional free text if respondents felt that their attitude or opinion was not offered in the choice options.

Open-ended questions were designed to understand the reasons behind some selected choices of closed-ended questions and to prompt respondents to reflect on their training and development as well as the challenges they face in diagnosing and treating patients’ conditions.

As this study aimed to understand the values and preferences of potential users of a pelvic simulator rather than their views of a specific simulator, no proposed solution was provided to avoid influencing respondents’ answers. Respondents were only informed that the simulator could be used for medical training, patient education and medical device testing.

The survey was pretested before dissemination by 1 urogynecologist, 1 pelvic medical device engineer, 1 junior doctor, and 2 researcher engineers. The pretesters were asked to provide feedback on the usability of the survey and its presentation, the clarity of the questions, and how long it took to complete. No suggestions were made on the content of the questions. The resulting feedback regarding the usability and readability of the survey was implemented accordingly. The responses from the pretest were not included in the study results.

### Ethical Considerations

Ethics approval was granted by the University of Bristol’s Faculty of Engineering Research Ethics Committee (reference number 13989). Respondents were required to complete the inbuilt consent form to take part in the survey. Respondents could withdraw their data from the study within 4 weeks of taking part, but none chose to do so. There was no financial compensation offered for taking part in the survey. Any identifyable data were stored separately from the analyzed data on an encrypted hard drive which only the main researcher had access to.

### Recruitment and Data Collection

The survey was originally designed for and advertised to professionals working in different areas of pelvic health. As a result, recruitment emails were sent to 2 professional networks of pelvic health practitioners for distribution among their members: the British Society of Urogynaecology [29] and Pelvic Obstetric and Gynaecological Physiotherapy [30]. Individual recruitment emails were sent to pelvic health practitioners with experience in medical education who were identified through their affiliated university profiles and organizations such as Lead Midwives for Education [31]. Pelvic floor medical device engineers and designers were recruited through their companies. Although the recruitment emails were targeted at United Kingdom—based professionals, those working abroad whether they had relocated or been forwarded the email by a colleague in the United Kingdom, were also encouraged to participate in the survey.

The recruitment email briefly explained the purpose of the study, signposted recipients to an attached 2-page document for further information and encouraged them to ask any questions relating to the study. Recipients were invited to complete the internet-based survey by following a URL link and were encouraged to forward the email to colleagues working in pelvic health.

The survey was hosted on Qualtrics (Qualtrics International Inc) and was available for completion for 3 months between May and August 2023 and for an additional month between March and April 2024. Respondents were advised that the survey should take roughly 10 minutes to complete and were notified that once the survey was begun, they would be able to return to it to complete at a later time if convenient.

### Data Analysis

Identifiable information such as email addresses, if provided, was removed before analyzing the data. Data from respondents who did not fully complete the survey were not included in the analysis and responses were excluded where there were fewer than 10 respondents from a given profession to ensure statistically meaningful results. Quantitative data were analyzed in Python (Python Software Foundation) using descriptive statistics such as the mean and SD. For rating scale data, the scales were mapped to a numerical scale: for example, not important=1, moderately important=2, and very important=3 to calculate the variables. Differences in results based on demographic groups were identified using 2 sample Kolmogorov-Smirnov 2-sided tests. Qualitative analysis was conducted in NVivo software (Lumivero) using thematic analysis [32]. Using this method, the principal researcher (YZ) identified themes after familiarization and initial coding of the responses to the open-ended questions. The themes were then refined by 2 researchers (YZ and AT) and codes were grouped into concepts relating to the themes.

## Results

### Demographic Data

In total, 146 people initiated the survey, and 85 surveys were fully completed, 66 of which were by physiotherapists. There were less than 10 respondents from other pelvic health professions which were excluded to focus on one core group’s needs and requirements. At the time of survey dissemination, there were 1219 active members of the Pelvic Obstetric and Gynaecological Physiotherapy who would have received the recruitment email which indicates a 5% response rate. With a 90% CI and 10% margin of error, an ideal sample size for a population of 1219 is 65 people [33].

Table 1 presents the demographic composition of the respondents included in the analysis. Most respondents were female (61/66, 92%), between 45 and 54 years of age (21/66, 32%), with more than 10 years of professional experience (44/66, 67%) working in the United Kingdom (62/66, 94%). Although an additional open ended demographic question on ethnicity was asked, we have not included the responses as the types of answers were too varied (eg, some answered based on ethnicity and others on ethnic category).

**Table 1.** Respondent demographics (n=66).

Demographics	Values, n (%)
Professional experience (years**)**	
Less than 5	8 (12.1)
5‐10	14 (21.2)
More than 10	44 (66.7)
Country of practice	
United Kingdom	62 (93.9)
Hong Kong	1 (1.5)
Australia	1 (1.5)
Prefer not to say	1 (1.5)
Sex	
Female	61 (92.4)
Male	1 (1.5)
Prefer not to say	4 (6.1)
Age range (years)	
25‐34	5 (7.6)
35‐44	20 (30.3)
45‐54	21 (31.8)
55‐64	17 (25.8)
65‐74	2 (3)

### Quantitative Results

#### Use Cases

Respondents were asked how they see themselves using a pelvic floor simulator in their practice. The most common use case is for training and professional development (n=56), followed by educating patients (n=48) and practicing examinations (n=44). Less common use cases of the simulator include as a research tool (n=16) and for medical device testing (n=11). Although 1 respondent said that they would not use a simulator, due to not seeing it as useful at the current stage of their career (specialist pelvic health physiotherapist), they added that it could be helpful for patient education. There was no significant difference between the respondents’ choices of use case and their professional experience (*P*>.49).

#### Simulator Features

**Figure 1.**
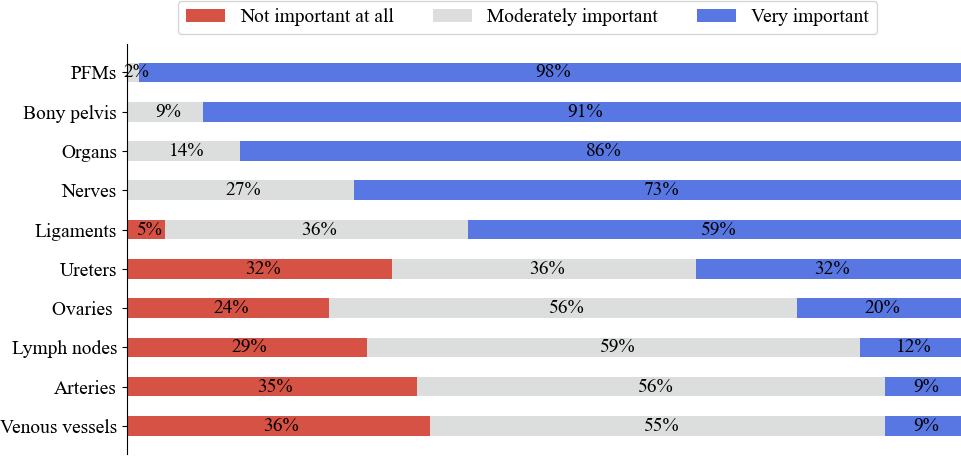
Rated level of importance of anatomical structures to be included in the simulator. PFM: pelvic floor muscle.

Respondents considered the PFMs, bony pelvis, pelvic organs (excluding the ovaries and fallopian tubes), nerves, and ligaments to be the most important anatomical structures to include in a simulator. Figure 1 illustrates the mean Likert rating of each structure alongside the raw data.

Out of the 6 presented options for functions and dysfunctions for the simulator to demonstrate, all were considered to be very or moderately useful. Table 2 presents the overall mean and SD between all respondents. Prolapse and muscle tone had the highest mean scores of 3 and 2.95, respectively. There was no significant difference between the respondents’ considered utility of the functions and their professional experience (*P*>.34).

Respondents were asked whether it would be useful for a simulator to be compatible with imaging modalities such as ultrasound. This feature could enable users to practice probe positioning and diagnostic skills. Table 3 shows that compatibility of the simulator with medical imaging is not of strong importance. On average, all modalities scored between 1: not at all useful and 2: moderately useful.

**Figure 2.**
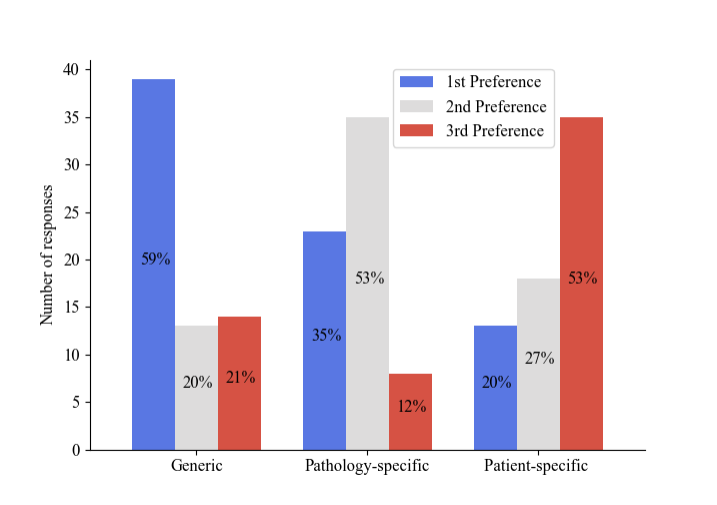
Stated preference for the specificity of the simulator.

Figure 2 illustrates the preference for a generic over a case-specific (pathology) or patient-specific simulator: a generic simulator was the most preferred (39/66, 59%) and a patient-specific simulator was the least preferred (35/66, 53%). Where respondents ranked the options equally, their preferences were counted for each option.

**Figure 3.**
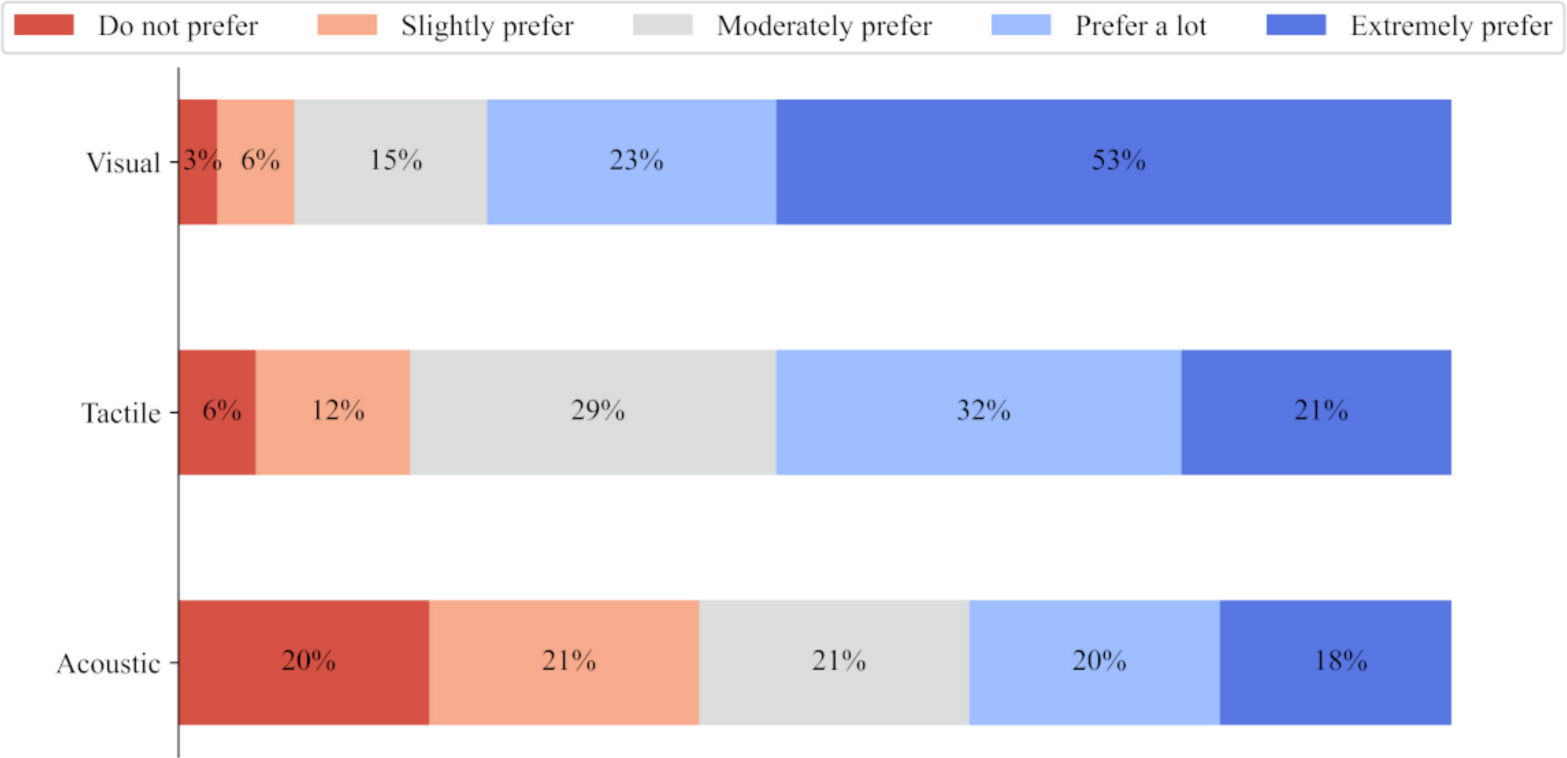
Preference for feedback modalities of the simulator’s sensors.

The preference between auditory, tactile or visual feedback modalities varied, as seen in Figure 3. Overall, visual feedback was most preferred (mean score 4.2, SD 3.8) and acoustic feedback was the least preferred (mean score 2.95, SD 2.8). There was no significant difference between respondents’ feedback preferences and their years of professional experience (*P*>.27) nor their age (*P*>.35).

Table 4 summarizes respondents’ preferences toward the modularity and sensing capabilities in a pelvic floor simulator as well as the term used to describe it. 54 respondents (54/66, 82%) agreed that there was some value in the simulator being modular, that is, for parts to be removable. In total, 82% (55/66) of respondents considered both the position and the pressure of a user’s interaction to be useful feedback through sensors embedded in a simulator. Overall, respondents preferred the term ‘simulator’ (43/66, 64.2%) over model, phantom or manikin.

**Table 2.** Utility of functions and dysfunctions to be demonstrated.^a^

Function or Dysfunction	Values, mean (SD)
Prolapse	3 (0)
Muscle tonicity	2.95 (0.21)
Intraabdominal pressure	2.94 (0.24)
Breath	2.9 (0.48)
Stiffness	2.82 (0.39)
Posture or gait	2.75 (0.43)

a Responses were on a 3-point Likert scale (1=not useful at all; 2=moderately useful; and 3=very useful*).*

**Table 3.** Importance of imaging compatibility.^a^

Imaging modality	Mean (SD)
Magnetic resonance imaging	1.3 (0.58)
Computed tomography	1.27 (0.54)
Fluoroscopy	1.24 (0.55)
Ultrasound	1.89 (0.76)

a Responses were on a 3-point Likert scale (1=not useful at all; 2=moderately useful; and 3=very useful).

**Table 4.** Respondents’ attitudes toward modularity, sensing, and terminology preferences for PFM simulators.

Questions and answers	Values, n (%)
How important is it for the pelvic floor simulator to be modular?	
Not at all important	12 (18.2)
Moderately important	29 (43.9)
Very important	25 (37.9)
What information from sensors would be useful to provide?	
Unsure	1 (1.5)
Positional tracking	2 (3)
Pressure applied	8 (11.9)
Position and Pressure	55 (82.1)
Which term do you prefer to describe a pelvic floor simulator?	
Simulator	43 (64.2)
Model	22 (32.8)
Phantom	0 (0)

### Qualitative Results

#### Overview

The most common areas where better training needs were identified were the assessment of muscle tone (normal contractions, hypertonicity, and tone variation) (n=27) and POP (n=22). The need for better vaginal (n=11) and rectal (n=10) examination training was also identified, as was the need for anatomical mapping skills (n=4), that is, feedback on which muscle is palpated internally. Other areas include levator ani avulsion (n=6), vulvodynia (n=4), dyssynergy (n=3), pessary fitting (n=3), vaginismus (n=3), and nerve dysfunction (n=3). In total, 7 respondents felt that there were no areas where better training was needed.

Table 5 presents the most common training needs with respect to the respondents’ years of professional experience. For example, 62.5% (5/8) of physiotherapists with less than 5 years of experience reported a need for better training in identifying and assessing POP. This was almost triple that of physiotherapists with over 10 years of experience (10/44, 22.7%). Although only 8 respondents had less than 5 years of experience, better training for pelvic examination (rectal and vaginal) and anatomy mapping skills were not mentioned by them.

Three main themes emerged from the thematic analysis of the open-ended questions, summarized in the following sections. The themes, subthemes and a subset of the respondents’ quotes are shown in Table 6. In the optional further comments, 9 respondents expressed positive sentiments and excitement regarding a dynamic pelvic floor simulator as a potential outcome of this research. A total of 13 respondents expressed concern over a potential simulator, however these were mainly to do with the cost and implementation and are included in the analysis in Section 3.3.4.

**Table 5.** The most common areas where needs for better training were identified per physiotherapists’’ years of professional experience.

Training needs	<5 years, n (%)	5‐10 years, n (%)	>10 years, n (%)
None	1 (13)	—^a^	6 (14)
Tone variation	3 (38)	9 (64)	15 (34)
Prolapse	5 (63)	7 (50)	10 (23)
Vaginal examination	—	2 (14)	9 (21)
Rectal examination	—	2 (14)	8 (18)
Levator Ani avulsion	1 (13)	1 (13)	4 (9)
Anatomy mapping	—	2 (14)	2 (5)

a Not available

**Table 6.** Emergent themes with a subset of the respondents’ quotes from thematic analysis of the open-ended questions.

Themes and subthemes	Quotes
Prioritizing patient care	
Simulators can reduce the burden on patients of learning ‘on-the-job’	“There could be better ways [than peer practice], as colleagues might not have dysfunctions, so one ultimately needs to learn often about those ‘on the job’” “[We need better training for] More simulated examples of what might be a cause for concern. I found it difficult in the beginning to identify prolapse.” “[We need better training for] Correct pelvic floor contraction (have to feel a lot before you work out what is normal).”
Simulators have value in medical training and for patient education	“I think this is a wonderful development for pelvic health training and development” “I am excited at the prospect of having access to a pelvic floor simulator”
Patient communication is as important as practical examination skills	“I learn as much from listening to my patients as the physical examination but I don’t think this aspect [of] treating someone is very well explored in many pelvic health education courses.” “[We need better training for treating] Neurodiverse people with potential differing requirements regarding treatment. This could be related to communication style, or environmental or anatomical sensitivities, which can (should) impact how treatment is performed.” “[We need better training for] Supporting people with learning difficulties or physical disabilities in fulfilling the potential for safe physical relationships.” “[We need better training for] Transgender people and how physiotherapists and other health care professionals can support their journey better.”
Simulators as supplements, not replacements to human models	“Pelvic floor muscle function assessment techniques need human volunteers for learning and practice” “Other online courses are available but in my experience are not as beneficial as practicing on a live model.”
Representing the variability in anatomy and PFDs^a^ characterizes simulator realism	
Need for simulators that are representative of anatomical variation and properties	“[We need better training for] Variations on ‘normal’” “[We need] More examples of anatomical differences” “[We need] More lifelike pelvic floor models. That show that tension in pelvic floor” “[We need] A dynamic pelvic floor muscle assessment model for both Vaginal and Anorectal assessment of the pelvic floor muscle function - ideally includes superficial and deep muscle activity”
Requirements of the simulator depend on the use case and user	“I don’t really need to know about the vasculature for my examinations” “A simple model would be better for teaching professionals new to the area of pelvic health. A more detailed model for more advanced practice or teaching.” “Patients wouldn’t be likely to need to see arteries/ venous vessels or lymph nodes”
Importance of implementation, cost and accessibility in the design of simulators	
Doubt in technological capability	“[I’m] unsure if any model could do this, for example, exam for prolapses” “There are no simulators or models that can mimic the dynamic parameters that are tested by physiotherapist via vaginal or anorectal examination.”
Practical importance of integration with health services	“cleaning accessibility [should be considered].” “As I work in the NHS^b^ I am just mindful of getting the simulator to work alongside current IT systems”
Simulator features depend on affordability	“It sounds ideal for teaching! However cost will be a big factor for NHS staff or departments” “Important it is not too complex and expensive so that it isn’t out of reach for physios to purchase. Yet sounds amazing if it can fulfill the previous suggestions” “It’s likely to be far too expensive for an NHS physiotherapy department to buy’'

a PFDs: pelvic floor disorders.

b NHS: National Health Service.

#### Prioritizing Patient Care

Value was found in high-fidelity PFM simulators both for medical students’ practice and for communicating with patients. Respondents reported that peer practice was the most common modality for practical training. However, as fellow colleagues may not present any of the variety of PFDs, respondents reported that diagnosing PFDs is learned “on-the-job” at the start of their careers. Although several respondents considered peer practice or using human volunteers as the best way to learn, respondents also indicated that an alternative option such as a simulator would be favorable.

The importance of communication skills was highlighted as much as the examination skills in treating a patient. Respondents mentioned difficulty communicating with patients or adapting to potentially differing needs of neurodivergent patients. While a simulator may not always be the answer in the context of improving patient communication, it may be useful as a tangible tool for some of the areas where respondents reported difficulties in their practice. For example, challenges in supporting differently abled patients in fulfilling safe physical relationships and considering sensitivities in examining survivors of sexual abuse or trauma. A simulator may assist a practitioner as a demonstrative tool in communicating with such patients.

#### Representing the Variability in Anatomy and PFDs for Simulator Realism

The use of simulators was the second most common practical training modality reported. However, respondents recognized that existing simulators lack realism. Specifically, pelvic models with no tension in the pelvic floor.

The anatomical structures to be included in a simulator were reported to vary depending on user and use case. This agrees with the aforementioned commonly shared opinion that the appropriate level fidelity is contingent on the type of task it is used for and the expertise of the user [4,7,8]. For example, if used to demonstrate the symptoms of a condition to patients, it would not need the lymph nodes, whereas these are important anatomical structures for oncologists. However, the same requirements were not shared by all respondents. Some considered vasculature to be important for patients, and others did not consider it to be relevant. Overall, respondents suggested that there should be less detail if the simulator is used to educate patients than if it is used for health care professionals. Some respondents thought no changes would be needed, while some considered this to be on a case-by-case basis.

#### Consideration of Implementation, Cost, and Accessibility of Simulators

The affordability of a simulator that could offer some of the features included in the survey was a shared concern amongst respondents, specifically for the United Kingdom’s National Health Service (NHS) departments. The challenge of logistical integration within the NHS computer systems was also noted. For example, if the simulator needed to be plugged in to a computer to provide visual feedback on a screen. Although no specific simulator was proposed in his study, 2 respondents had low faith that any simulator or model could offer some of the features mentioned.

### Design Requirements

In total, 6 recommendations for the design of future pelvic floor simulators have been formulated as a result of the survey findings and are displayed in Table 7.

**Table 7.** Design recommendations elicited from survey responses.

Design recommendation	Explanation
Realistic materials	Materials with similar properties to human muscle and skin, such as silicone should be used to provide a high level of fidelity.
Demonstrates prolapse and muscle tone	A simulator should at least demonstrate increased, normal and reduced PFM^a^ tone for example, with a material that has stiffness-changing properties which users can apply resistance as if assessing actual muscle tone. This would also allow medical devices such as PFM trainers to be evaluated for their ability to differentiate between increased, normal or reduced tone.
Modular in design	A simulator should comprise adaptable anatomical complexity for patients and professionals with different experience levels
Position and pressure sensing	The palpation location should be tracked, and forces applied should be measured, both to be displayed and fed back to the user
Range of feedback modalities	Feedback modalities should be customizable according to preference. Feedback would be useful for supervisors or teachers and observers to better understand (ie, see and hear) the interactions between the simulator and the user, who would be the only one receiving tactile feedback.
Affordable and accessible	A simulator should be affordable and be designed in line with the relevant curriculum to maximize the number of people able to access it.

a PFM: pelvic floor muscle.

## Discussion

### Principal Findings

The results from our survey agreed with and built on previous research on the design and use of 3D pelvic simulators and models. All but one of the respondents reported that they would use a pelvic floor simulator for at least one use case. In addition, respondents held strong value in the simulator as a tool for demonstrating pelvic floor function and dysfunction which supports the existing literature [15,21,23].

The anatomical structures rated of high importance, illustrated in Figure 1, confirm those found by Meyer et al [18], namely, the bones, muscles, organs, and nerves. However, the experts consulted in their Delphi study considered some ligaments to be unimportant to include, and ovaries and veins as important, with the opposite being the case in our results. This discrepancy may be explained by the difference in health care professionals consulted in their study (basic scientist, obstetrics and gynecology clinician, and urologist). For example, ovaries are not always easy to palpate in a pelvic examination if a patient is overweight or unless there is a cyst or dysfunction [34]. As a result, the inclusion of ovaries may be of less importance to physiotherapists in the context of skill-based training than would be the case for gynecologists who would need to refer to these structures in surgical training [35]. Commercial pelvic simulators demonstrate this distinction, for example, the surgical [36] simulator from limbs and things includes the ovaries whereas the clinical simulator [37] does not.

As aforementioned, the level of fidelity depends on the skills being taught and the intended users [4,7,8]. High-fidelity simulation is often associated with high cognitive load and may be better suited for more experienced trainees or continuing professional development. This was echoed in our results whereby respondents suggested the simulator would need different levels of complexity depending on the user and considered modularity as a useful feature, which lead to including modularity as a design recommendation. Removing certain anatomical structures through a modular function has also been suggested in the evaluation of a virtual PFM anatomy simulator [38].

Learning from GTAs or through peer practice is considered the best form of training for learning and practicing the pelvic examination [39,40]. Training with GTAs has been shown to improve confidence and decrease anxiety for performing examinations in clinical settings [41], as has training with simulators [42]. Respondents mirrored this whereby it was reported that although peer-practice provides the most realism, an alternative would be beneficial. Pelvic simulators can be integrated into the curriculum alongside GTAs or peer practice, providing a supplementary tool that can offer repeated practice while reducing both the risk of causing harm to a peer or GTA and medical students’ fear of causing it [43,44]. This evidence supports the design recommendation of using realistic materials and materials with smart properties to provide higher fidelity.

Feedback in medical simulators has been identified as one of their most valuable features [45,46]. Similar to the feedback that would be received through GTA or peer practice, feedback from sensors embedded in simulators can provide the information required to conduct the examination effectively. Figure 3 shows that the preferred modes of feedback from sensors were foremost through a visual display and second, by a tactile vibration or applied pressure which have been previously reported as the 2 commonly preferred modalities of urologists [47]. In physical examinations, physiotherapists rely on a combination of tactile, visual, and acoustic information. For internal examinations, tactile feedback would be the most realistic to integrate into a simulator as visual and acoustic cues would be more indicative of a patient’s facial and uttered expressions to signal discomfort [48]. While the use of feedback is included as a design recommendation, it would be important to consult with end-users during the design process to optimize how this feature is integrated. Without mentioning the price of a potential simulator in the survey itself, several respondents expressed concern over the cost and resulting lack of accessibility or included functionalities. Given that most respondents were working in the United Kingdom at the time of participating in the survey, there was specific mention of NHS departments being unable to afford a simulator that offered such features. It may be that some of the questions were answered with this consideration in mind. However, a study of over 400 final year medical students in the United Kingdom found that simulator-based teaching is less expensive per student for pelvic examination training than GTA-led teaching [49].

Certain features, such as sensors which were of interest to users, may increase the cost of a simulator beyond budget constraints despite being included as a design recommendation. Through careful design and consultation with end-users, a balance can be struck between cost and fidelity, ensuring the simulator provides high-quality, curriculum-relevant training while remaining affordable and accessible.

As a tool for patient education, the use of 3D models has proven efficacy in enhancing communication with patients [50], ensuring young patients’ parents understand their conditions [51,52], and improving the attitudes toward pelvic examination by teaching nonmedical professionals how to perform the examination [53]. The survey results indicated that patient education was the second most popular use case of a pelvic floor simulator, with respondents highlighting several ways in which it could be used to assist in specific conditions. At later design stages, it would be beneficial to include patients in the design process as potential end-users themselves.

### Limitations and Further Work

Further work should explore other pelvic health experts’ opinions in equal detail and widen the participation to other countries as well as to patients with PFDs, who could also be users of such a simulator. For simplicity, anatomical structures were grouped to reduce the length of the survey. In the design stages of a given simulator, the importance of including some individual anatomical structures should be considered. For example, the pudendal and sciatic nerves are important structures that can cause pelvic pain when damaged whereas smaller, less significant nerves may be less relevant for a given use-case.

### Conclusions

Our study highlighted the value of dynamic and high-fidelity pelvic floor simulators for pelvic health physiotherapists in their own education and as a tool to aid communication with their patients. The primary outcome was 6 recommendations for the design of future dynamic pelvic floor simulators, specifically for physiotherapists needs. Our findings revealed that while there is a strong need for educational tools to supplement the pelvic health curriculum, the cost of such innovations is an important factor that needs to be considered to ensure they are accessible to those who can benefit from them.

This contribution provides pelvic simulator designers with the knowledge of the needs and concerns of a crucial target user group which can better inform their product development. The importance of end-user involvement at each stage of the design process is paramount to ensuring such tools will serve the community of pelvic physiotherapists and their patients.

## Supplementary material

10.2196/72119 Multimedia Appendix 1 Questionnaire questions and answer formats.

## Abbreviations

GTAs: Gynecological Teaching Associates
NHS: National Health Service
PFD: pelvic floor disorder
PFM: pelvic floor muscle
POP: pelvic organ prolapse
POP-Q: pelvic organ prolapse quantification
