# Supplementary material for: Requirements and Value Elicitation for a High-Fidelity Pelvic Floor Simulator for Physiotherapists: Mixed Methods Study
Source: JMIR Hum Factors. 2025 May 15;12:e72119. doi: 10.2196/72119 (PMC12097285; doi:10.2196/72119)
Supplement: Multimedia Appendix 1 [file humanfactors-v12-e72119-s001.docx]

1. What is your job title?

|  |
| --- |

1. How many years have you been working in a field related to pelvic health?

- less than 5 years
- 5 to 10 years
- more than 10 years
- I do not work in Pelvic Health

1. What is taught in your pelvic health training and how is it currently taught? Or, if you are an educator, what do you teach and how?

What are the main items on the curriculum and what tools, simulators, mannequins, books or video material are used?

|  |
| --- |

4. Are there any examinations or dysfunctions related to the pelvic floor that you think there should be better training for?

|  |
| --- |

5. How do you see yourself using a pelvic floor simulator in your profession?

The use-cases you choose will be explored in the subsequent questions.

- I wouldn't use one
- Practicing examinations
- Training and professional development
- Surgical planning
- Patient education
- Medical device testing
- Research tool
- Other(s) please specify

|  |
| --- |

(if selected ‘Medical device testing’ in Q5)

5.0. You selected using the simulator to test a medical device.

If you have developed medical device, how do you currently test it?

|  |
| --- |

(if selected ‘I wouldn’t use one’ in Q5)

5.1. Why would you not use a pelvic floor simulator in your practice?

|  |
| --- |

6. Considering the use case(s) you identified in the previous question, how important is the inclusion of the following anatomy in the simulator?

|  | Not important at all | Moderately Important | Very Important |
| --- | --- | --- | --- |

**Pelvic floor musculature** (levator ani, coccygeus, piriformis, obturator internus, external urethral and anal sphincters, superficial transverse perineal, deep transverse perineal, bulbospongiosus and ischiocavernosus)

**Bony pelvis** (sacrum, coccyx, ilium,

ischium, and pubis)

**Pelvic floor organs** (bladder, rectum, vagina and uterus with cervix)

**Ovaries** (with fallopian tubes, ovarian ligament and suspensory ligament of ovary)

**Ureters**

**Ligaments** (parametrium, symphysis, and round ligaments)

**Arteries** (aorta, uterine, internal and external iliac arteries)

**Venous vessels** (Iliac veins)

**Nerves** (pudendal, superior and inferior hypogastric plexus)

**Lymph nodes** (pelvic and para- aortic)

Other(s), please specify

(if selected multiple use cases in Q5)

6.1. You envisioned using the pelvic floor simulator for multiple use cases. How do you think the requirements chosen in the previous question differ depending on the use case?

|  |
| --- |

1. The pelvic floor has numerous functions and can have many dysfunctions.

Which of the following would be useful for the simulator to demonstrate?

|  | Not useful at all | Moderately useful | Very useful |
| --- | --- | --- | --- |

**Muscle hypertension vs. muscle hypotension**

(i.e. to demonstrate pelvic floor exercises and causes/symptoms of incontinence and pelvic pain)

**Pelvic organ prolapse**

(Prolapse of the bladder, urethra, uterus, vagina or rectum)

**Intra-abdominal pressure**

(i.e. coughing, sneezing or carrying a load)

**Impact of posture or gait**

(i.e. sit-stand-supine or walking movements)

**Impact of breath on pelvic floor** (i.e.hypopressive and valsalva exercises)

**Effect of stiffer muscle tissue on pelvic floor**

(e.g. scar tissue and adhesions from an episiotomy, endometriosis or damage from mesh surgery)

Other, please specify:

1. Sensors embedded in the simulator can monitor your interaction with it and provide feedback to you.

Considering the use cases and features you identified in the earlier questions, what information from sensors would be useful to provide?

*Select all that apply*

Positional tracking of the user's interaction

Pressure applied by the user at a given point

Other(s), please specify:

1. When interacting with the simulator, how would you like feedback to be provided to you?

Feedback in this context is what you hear/see/feel when interacting with the simulator.

*Drag the slider for each option to record your preferences*

|  | Do not prefer | Prefer slightly | Prefer a moderate amount | Prefer a lot | Prefer a great deal |
| --- | --- | --- | --- | --- | --- |
|  | 1 | 2 | 3 | 4 | 5 |

Auditorily through a speaker

Tactile through a vibration or applied pressure

Visually through a screen

Other, please specify:

1. How important is it for the pelvic floor simulator to be modular or for parts to be removable?

- Not at all important
- Moderately important
- Very important

1. How important is it for the simulator to be compatible with the following imaging equipment?

|  | Not important at all | Moderately Important | Very Important |
| --- | --- | --- | --- |

MRI

CT

Fluoroscopy

Ultrasound

Other(s), please specify:

1. Is it preferable for the simulator to be patient-specific, case-specific or generic?

*Enter your preference from 1 (most preferred) to 3 (least preferred). If any of your preferences are the same, you can enter the same number in those boxes.*

Patient-specific (produced from an imaging scan of an individual)

Case-specific (to resemble certain pathologies)

Generic (based on the anatomy of the average individual)

1. Which term do you prefer to describe a pelvic floor simulator?

High-fidelity pelvic floor ___________

- Phantom
- Simulator
- Model
- Manikin
- Other, please specify:

14. Do you have any further comments relating to the content of this questionnaire?

15.Would you be available for a follow-up conversation at your preferred time and place/online?

- If yes, enter email here
- No

Before you submit your answers, please take a moment to answer these 4 questions to help us monitor the representation of the questionnaire respondents.

1. In which country do you work as {Insert answer from Q1}?
2. What is your age?

- Under 25
- 25 – 34
- 35 – 44
- 45 – 54
- 55 – 64
- 65 – 74
- Over 74

1. How do you describe yourself?

- Male
- Female
- Non-binary / third gender
- Prefer to self-describe
- Prefer not to say

1. What is your ethnic origin?
